# The importance of partnership in chronic pain – co-creation of tomorrow's pain care by working together in research, implementation, and knowledge dissemination

**DOI:** 10.3389/fpain.2026.1765233

**Published:** 2026-03-09

**Authors:** Paulin Andréll, Birgit Heckemann, Anna Grimby-Ekman, Gunilla Göran, Marie-Louise Olsson, Roger Johansson, Paula Forslund, Axel Wolf

**Affiliations:** 1 Department of Anesthesiology, Intensive Care Medicine and Pain Medicine, Sahlgrenska University Hospital, Gothenburg, Sweden; 2 Department of Anesthesiology and Intensive Care Medicine, Institute of Clinical Sciences, Sahlgrenska Academy, University of Gothenburg, Gothenburg, Sweden; 3 Institute of Health and Care Sciences, Sahlgrenska Academy, University of Gothenburg, Gothenburg, Sweden; 4 School of Public Health and Community Medicine, Institute of Medicine, Sahlgrenska Academy, University of Gothenburg, Gothenburg, Sweden; 5 The Swedish Rheumatism Association, Stockholm, Sweden; 6 The European Network of Fibromyalgia Associations, Stockholm, Sweden; 7 The Swedish National Association for Fibromyalgia and Chronic Pain, Västra Frölunda, Sweden; 8 Patient Representative at Living Library [Levande Bibliotek], Gothenburg, Sweden; 9 Institute of Nursing and Health Promotion, Oslo Metropolitan University, Oslo, Norway; 10 University of Gothenburg Centre for Person-Centred Care (GPCC), Sahlgrenska Academy, University of Gothenburg, Gothenburg, Sweden

**Keywords:** chronic pain, partnership, patient and public involvement, person-centered care, societal impact of pain

## Abstract

Chronic pain is a long-term condition that affects the daily lives, identities, and well-being of millions of people worldwide. Effective pain care remains limited. Moreover, lack of knowledge and awareness among healthcare professionals and patients, combined with the invisibility of pain, often leads to disbelief, delayed diagnosis, prolonged suffering, and significant societal costs. Novel approaches are necessary to improve pain care, as most countries’ healthcare systems are struggling to provide adequate support for persons living with chronic pain. This article highlights the importance of partnership in chronic pain management. We present examples from Sweden that demonstrate the benefits of stakeholder collaboration to improve pain care. Partnership between patients and healthcare professionals is a core element of person-centered care (PCC). We present PCC as a necessary strategy that combines ethical commitment with demonstrable clinical and economic benefits. We highlight the unique value of patient and public involvement (PPI) in pain research and innovation, where patients with lived experience can help researchers identify deficiencies in pain care and service provision, formulate relevant research questions, and develop projects that address real needs and priorities. Finally, we provide concrete examples of successful cooperation between patients, healthcare professionals, patient associations, and researchers in raising awareness of chronic pain and translating research findings into clinical practice. Collaboration among healthcare professionals, patient associations, and researchers is essential to make the best use of knowledge and lived experience to co-create and implement effective pain care, advance research, and raise awareness of chronic pain in healthcare and society.

## 1 Introduction

Almost 20% of the European population live with moderate-to-severe chronic pain (1). Prevalence of chronic pain increases with age (2), affecting many people in midlife and often interfering with work (3). Despite affecting millions of people, poor management of chronic pain is a massive burden on society, with low back pain being the leading cause of years lived with disability worldwide (4). Contributing factors include inaccessible pain care, lack of adequate pain diagnosis and treatment, as well as insufficient knowledge among healthcare professionals and patients (5). Although vast evidence demonstrates the benefits of pain rehabilitation in enhancing clinical outcomes for persons living with chronic pain, its implementation in clinical practice remains limited. There is a need for new approaches to address these issues and improve pain care, as today's healthcare system is insufficient to support people living with chronic pain. In this perspective article, we highlight the importance of stakeholder partnerships in chronic pain management and present examples from Sweden that demonstrate the benefits of collaboration across multiple sectors to innovate and improve pain care.

## 2 Partnership in person-centered care - clinical evidence and implications

Chronic pain and other chronic conditions remain complex challenges in modern healthcare. Biomedical approaches alone are insufficient to address their multidimensional impact, which extends beyond symptoms and affects daily functioning, identity, relationships, and social participation. Person-centered care (PCC) has therefore gained recognition as a necessary strategy that combines ethical commitment with demonstrable clinical and economic benefits. The partnership between patients and healthcare professionals is a core element of PCC.

The University of Gothenburg Centre for Person-Centred Care (GPCC) has developed a systematic framework integrating ethics with clinical practice (6), it is among the most used frameworks in controlled trials of PCC. The framework's philosophical foundation is rooted in personalism, particularly the work of Ricoeur and Mounier (7), which emphasizes dignity, agency, vulnerability, and relationality. Thus, it can be considered a structured method for enacting ethical principles in everyday care, rather than an abstract ideal.

The GPCC framework is based on three interconnected processes: (1) eliciting the patient's narrative, (2) establishing a partnership based on a shared health plan, and (3) documenting the plan to ensure transparency and continuity (8).

Quantitative and qualitative evidence support the effectiveness of PCC, including shorter hospital stays, improved self-efficacy, enhanced symptom control, better health-related quality of life, improved physical function, and cost savings. For instance, in the PCC-HF (heart failure) trial, systematic follow-up of a person-centered health plan during hospitalization reduced mean length of stay by 30% and improved activities of daily living among patients with chronic heart failure (6). Systematic reviews have identified similar benefits in cancer, mental health, inflammatory bowel disease, and chronic pain in a variety of settings, suggesting broad applicability across clinical contexts (6, 9). Qualitative studies emphasize the importance of partnership in practicing the ethics of PCC – adherence to the GPCC framework is not achieved through rigid routine adherence, but rather through the consistent application of its ethical principles (6). The relevance of partnership is particularly pronounced in chronic pain care. Patients with persistent pain often report that their experiences are overlooked by clinicians. Eliciting the patient's narrative enables clinicians to align interventions with personal goals, such as maintaining employment, engaging in family life, or managing anxiety. The co-created health plan promotes self-management and autonomy, while documentation strengthens continuity of care across settings. In digiphysical contexts, where digital and face-to-face encounters are combined, these principles have been shown to improve patient engagement and communication. Evidence from five consecutive GPCC studies conducted in inpatient, outpatient, primary care, and home-based settings affirms the effectiveness of the GPCC framework in physical as well as digital environments (10). These studies showed consistent improvements in patient-reported outcomes, functional ability, and quality of life, which were accompanied by reductions in healthcare utilization in several cases. These results demonstrate that the GPCC framework is robust across diverse levels and modes of healthcare delivery. Health economic assessments further strengthen the case for PCC. A recent systematic review of economic evaluations showed that PCC is often either cost-saving or cost-effective compared to standard care (11). In many evaluations, PCC “dominated” standard care by delivering better outcomes at lower or equivalent cost. Where costs were higher, the incremental cost per quality-adjusted life year (QALY) remained within accepted thresholds. These findings suggest that investment in PCC infrastructure, such as staff training, documentation systems, and digital tools, is justified by improved outcomes and system-level sustainability.

The implications of PCC for chronic pain care are multi-level. For patients, partnership translates into improved self-efficacy, reduced uncertainty, and a better quality of life. For clinicians, PCC fosters more accurate assessments, stronger therapeutic relationships, and enhanced job satisfaction (12). For organizations, structured PCC can support coordination across services, reduce duplication, and shorten hospital stays. At a societal level, partnership-based PCC contributes to more sustainable and equitable healthcare, as functional improvements and enhanced quality of life reduce both direct costs and broader socio-economic burdens.

However, challenges remain. Existing trials have relied on outcome measures defined by researchers rather than patients, which risks misalignment with the ethical foundation of PCC (6). Future research should adopt goal-attainment scaling and incorporate person-centered outcomes. Organizational structures and governance also require strengthening, including adequate training, supportive documentation systems, and leadership commitment. To create a system that addresses the unmet needs of persons living with chronic pain, we need to move beyond co-creation and strive for co-integration with all relevant stakeholders in the healthcare ecosystem. Thus, we need to transition from working in a linear and transactional fashion to a person-centered ecosystem approach that builds on continuity, trust, and relationships. Without these conditions, the partnership among stakeholders risks being episodic rather than systematic.

In the next section, we describe the importance and benefits of involving patients in pain research and innovation efforts.

## 3 Partnership in research and innovation – the importance of patient and public involvement

Patient engagement is the “active, meaningful, and collaborative interaction between patients and researchers across all stages of the research process, where research decision making is guided by patients’ contributions as partners, recognizing their specific experiences, values, and expertise” (13).

Patient and public involvement (PPI) in research and innovation promises unique value: people with lived experience of health conditions, direct use of care services, or a user's view of the healthcare system are key to identifying deficiencies in care and health service provision. In the context of chronic pain, PPI can provide a deeper understanding of the lived experience of persons living with chronic pain and their carers, as well as inequalities in pain care. PPI can hence enable researchers to formulate relevant research questions and develop projects that address real needs and priorities, strengthening both the quality and the relevance of pain research and innovation projects (14).

Over the past two decades, the importance of PPI for improving the quality and effectiveness of healthcare processes has been recognized internationally. In a 2023 framework for meaningful engagement, the World Health Organization stressed: “The right to participate is an essential feature of the right to the highest attainable standard of health. People have the right and duty to participate individually and collectively in the planning and implementation of their health and wellbeing” (15). This was further emphasized in the 2024 update of the Declaration of Helsinki: “*Researchers should enable potential and enrolled participants and their communities to share their priorities and values; to participate in research design, implementation, and other relevant activities; and to engage in understanding and disseminating results*” (16). In addition, involving patients in research planning and implementation is stated as a key objective of the European Pain Federation's (EFIC) Pain Research Strategy 2025 (17).

In practice, PPI is gradually becoming a key feature of healthcare systems as patients and the public are increasingly involved in shaping healthcare at the micro, meso, and macro levels, in policy and practice (18). In many countries, including the United States, Canada, Australia, the UK, and the Netherlands, PPI is part of public policy. In Sweden, PPI even has a legal foundation, namely the Swedish Patient Act (19), a legal framework that promotes patient integrity, self-determination, and participation in care decisions. Moreover, a major healthcare system transition focusing on “person-centered integrated care” is underway. “Person-Centered integrated care” is an agreement between the Swedish Association of Local Authorities and Regions and the Swedish government. It centers on organizing and delivering healthcare based on patients' needs and health conditions and prioritizes coordinated, person-centered, and importantly, co-created care (20). The transition entails a paradigm shift towards person-centered integrated care, where patients and healthcare professionals become equal partners in health research and clinical innovation and implementation (21, 22).

Funders and policymakers are increasingly mandating or prescribing equality and participation of patients and the public in decision-making. The National Institute for Health and Care Research (NIHR), UK Research and Innovation (UKRI), the Wellcome Trust, and the European Commission (especially for public procurement initiatives) have all made PPI a mandatory criterion in grant application evaluations. Hence, PPI has also become a cornerstone of health research and innovation (23), marking a radical change in how research and innovation projects are designed and conducted. Traditionally, researchers and healthcare professionals determined research and innovation agendas, and patients were seen primarily as study “subjects” whose role was restricted to providing data (24). Today, recognition has grown of the need to make research and innovation more democratic. Through PPI, patients and their carers can provide unique expertise that is rooted in their lived experience and shape how research questions are framed, interventions are designed, results are interpreted, and solutions to care problems are developed (24–26).

A key justification for PPI is that it strengthens democracy (27), by including the experience and expertise of citizens who use and pay for healthcare services or publicly funded research (28). Democratic processes that foster the inclusion of multiple perspectives and experiences can also lead to better decision-making (27). PPI inclusion is possible in every phase of the research cycle, from formulating research questions through to the implementation and dissemination of findings (29). Involving patients, family carers and/or the public can improve the relevance of research and provide different and valuable perspectives (30–32). However, effective PPI requires consideration and clarity about the purpose, methods, and expected level of participation. A recurring concern in the discourse about PPI is tokenism. People are invited in for consultation, but their influence remains limited, and the real decision-making power remains with the researchers. Thus, PPI can become a symbolic gesture of inclusion and mimic participation while leaving traditional hierarchies intact (33). Such practices risk eroding trust and diminishing the value of lived experience. Researchers need to carefully consider when PPI is needed, and what level of meaningful and democratic participation is appropriate at each phase. The representativeness of PPI is another crucial concern. Research teams need to strive for a balanced perspective within the project team and assess whether to engage individuals with personal lived experience, or patient associations that represent diverse views of a broader segment (34).

In summary, involving patients and the public as equal partners in research and innovation has the potential to strengthen democracy in healthcare. Yet, researchers and clinicians must be prepared to invest the time and effort to grow fruitful collaborative relationships.

In the next section, we discuss how healthcare professionals, researchers, and patients can work together to change pain policies on a societal level.

## 4 Partnership in societal impact of pain - working together to raise awareness of pain

Societal Impact of Pain (SIP) Sweden is one of twelve national platforms in SIP Europe, a multi-stakeholder partnership led by the European Pain Federation (EFIC) and Pain Alliance Europe (PAE), where patients, researchers, and healthcare professionals work together to raise awareness and knowledge about pain, improve pain care, and change pain policies (35, 36). SIP Europe has four main long-term priorities:

Integrated pain management
Pain in employment and economic considerations
Promoting pain research and education
Recognition for pain as a chronic condition

Chronic pain has a major impact on peoples’ lives affecting work ability (3, 37). Absence from work (sick leaves and early retirement) constitutes the largest component of the societal costs related to chronic pain (38). Musculoskeletal pain is also the most common reason for visits to general practitioners by frequent attenders in primary care in Sweden (39). Few are aware that chronic pain is a disease, and even fewer know what they can do to prevent pain from becoming chronic or recurring, or to manage the comorbidities associated with chronic pain. There is a lack of recommendations regarding self-care for persons living with chronic pain, and there is no established health coach support or specialist nurses for patients with chronic pain in Sweden. Furthermore, patients report challenges beyond healthcare in contacts with employers, social security, and employment services, and not being justly treated in society in many different situations, for example, in conversations with unsympathetic employers, colleagues, relatives, and friends (5, 40, 41). In contrast, people seeking care for hypertension or diabetes rarely describe that their physician lacks adequate knowledge to treat their disease, nor that their family members and employers are unaware that hypertension or diabetes is a disease. The inequalities evident in healthcare mainly stem from a lack of knowledge about chronic pain and the lack of recognition that chronic pain is a disease in its own right.

In Sweden, patient associations, healthcare professionals, and researchers have addressed the lack of pain care and insufficient knowledge of chronic pain by developing “The Swedish healthcare pathway for adults with chronic pain - a person-centered and coherent care (P3C) pathway” (5). The P3C pathway emphasizes the need for coherent, evidence-based and personalized delivery of healthcare, including early pain analysis, a biopsychosocial approach to assessment and treatment, an early rehabilitation plan, dialogue and joint plans between levels of care, patient participation, and education on pain and its consequences. The aim of the P3C pathway is to provide equal, accessible and evidence-based pain care across Sweden, where the initial biopsychosocial pain analysis, screening for risk factors for development of chronic pain and treatment (including rehabilitation) of pain should be performed in primary care.

The development of the P3C pathway included mapping of the current situation from both the healthcare and the patient perspectives. During this process, patient representatives described experiencing a lack of empathy during healthcare contacts and mistrust in the healthcare system, stressing that knowledge about chronic pain and its management is limited among both healthcare professionals and patients. They also called attention to the lack of a holistic approach and insufficient support for returning to a life with good management of both pain and its consequences. These barriers often lead patients to seek solutions outside the healthcare system, as they perceive a lack of knowledge among healthcare professionals. However, such solutions are frequently not evidence-based and, in the worst cases, may create additional health problems. To address these issues, the PC3 pathway promotes patient empowerment and equality in care, dialogue between patients and practitioners, self-management of pain instead of prolonged medical intervention, and increased knowledge about chronic pain, based on existing evidence and experiences. Cooperation brings together different perspectives, experiences, and the necessary knowledge to implement evidence-based pain care and increase awareness of chronic pain in the healthcare system.

SIP Sweden has identified three main priorities for the upcoming years to improve pain care nationally:

### 4.1 Promoting implementation of the P3C pathway and ICD-11

One of SIP Sweden's aims is to promote the implementation of the P3C pathway and the International Classification of Diseases (ICD-11) by arranging workshops and educational events for both healthcare professionals and patient associations. These efforts seek to ensure that chronic primary pain is recognized and that all affected persons can get a timely diagnosis and the right support from the healthcare system.

### 4.2 Facilitating research collaboration

Patient associations and researchers have identified a need to facilitate connection routes to enhance collaboration in pain research. Thus, SIP Sweden aims to create a “research hub” facilitating contact between patient associations and researchers. Working together from the start of a project ensures that the research is relevant from both the medical and patient perspectives and that the study design is feasible and adapted to participants with chronic pain and associated comorbidities. In these efforts, patient associations are also crucial for the dissemination and implementation of research findings.

### 4.3 Increase knowledge about pain among healthcare professionals and in society

SIP Sweden's most important aim is to promote knowledge exchange and education about pain for healthcare professionals, patients, and the general public. SIP Sweden launched the “European Day on Pain Awareness” in Sweden by hosting a webinar on “Chronic pain and mental health – the associations you need to know about” for all associations in SIP Sweden.

Developing and delivering pain education remains an important task for universities in Sweden and across Europe. As standardized education is a prerequisite for delivering equal pain care, SIP Sweden will also support teaching staff to improve nursing and medical undergraduate and graduate education across Swedish universities.

## 5 Discussion

Until recently, patient associations, researchers, and healthcare professionals have advocated separately for improved pain care. To raise visibility of chronic pain and to improve knowledge of chronic pain and its consequences for both healthcare professionals and in society, we see an urgent need for increased cooperation and collaboration between stakeholders. The development of the Swedish P3C pathway is a successful example that highlights the importance of ongoing collaboration to advance research, implement evidence-based care, and disseminate new knowledge to persons living with chronic pain (5). By working together to improve pain care by early assessment and management in primary care, chronification of pain and its consequences might be prevented. Furthermore, improved collaboration between stakeholders to support persons with chronic pain re-entering the workplace after sick leave is warranted (40, 42).

The GPCC framework provides a robust, evidence-based model for operationalizing partnership in person-centered care. The growing body of evidence, including clinical outcomes, qualitative findings, and health economic analyses, demonstrates that partnership is an ethical imperative, as well as a clinically effective and economically sustainable strategy. In the management of chronic pain and other chronic conditions, partnership must be recognized as a standard of care that is central to the future of health systems.

Involving patients and the public as equal partners in research and innovation has the potential to strengthen democracy in healthcare and is increasingly mandatory, reflected in EFIC's research strategy 2025 and the updated declaration of Helsinki 2024 (16, 17). PPI aims to improve the relevance and quality of publicly funded research and innovation efforts by taking into account the lived experience of patients and the public and addressing their specific needs.

To increase knowledge and awareness of pain, a joint effort is required. Together, we must strive for evidence-based pain care that places partnership at its core, provides interventions that strengthen patients' self-efficacy, and empowers patients to self-manage pain in addition to fundamental medical pain care, rehabilitation, manual therapies (43), and support.

## 6 Conclusion

Partnership is a prerequisite and a way forward for advancing chronic pain care. Healthcare professionals, researchers, and patient associations must work in collaboration to co-create, implement, and integrate effective pain care, conduct relevant and innovative research, and raise awareness of pain in healthcare and society.

## Data Availability

The original contributions presented in the study are included in the article/Supplementary Material, further inquiries can be directed to the corresponding author.
